# Wood Formation Modeling – A Research Review and Future Perspectives

**DOI:** 10.3389/fpls.2022.837648

**Published:** 2022-03-23

**Authors:** Annemarie H. Eckes-Shephard, Fredrik Charpentier Ljungqvist, David M. Drew, Cyrille B. K. Rathgeber, Andrew D. Friend

**Affiliations:** ^1^Department of Geography, University of Cambridge, Cambridge, United Kingdom; ^2^Department of History, Stockholm University, Stockholm, Sweden; ^3^Bolin Centre for Climate Research, Stockholm University, Stockholm, Sweden; ^4^Swedish Collegium for Advanced Study, Uppsala, Sweden; ^5^Department of Forest and Wood Science, Stellenbosch University, Stellenbosch, South Africa; ^6^Université de Lorraine, AgroParisTech, INRAE, SILVA, Nancy, France; ^7^Swiss Federal Research Institute for Forest, Snow and Landscape Research WSL, Birmensdorf, Switzerland

**Keywords:** wood formation models, tree growth, terrestrial carbon cycle, dendroclimatology, forestry, growth–climate interactions, xylogenesis

## Abstract

Wood formation has received considerable attention across various research fields as a key process to model. Historical and contemporary models of wood formation from various disciplines have encapsulated hypotheses such as the influence of external (e.g., climatic) or internal (e.g., hormonal) factors on the successive stages of wood cell differentiation. This review covers 17 wood formation models from three different disciplines, the earliest from 1968 and the latest from 2020. The described processes, as well as their external and internal drivers and their level of complexity, are discussed. This work is the first systematic cataloging, characterization, and process-focused review of wood formation models. Remaining open questions concerning wood formation processes are identified, and relate to: (1) the extent of hormonal influence on the final tree ring structure; (2) the mechanism underlying the transition from earlywood to latewood in extratropical regions; and (3) the extent to which carbon plays a role as “active” driver or “passive” substrate for growth. We conclude by arguing that wood formation models remain to be fully exploited, with the potential to contribute to studies concerning individual tree carbon sequestration-storage dynamics and regional to global carbon sequestration dynamics in terrestrial vegetation models.

## 1. Introduction

Wood formation and its interaction with the environment are of great relevance for a multitude of disciplines. For example, the value of wood as a raw material is of key interest for forestry as well as increasingly as bioenergy fuel (Downes and Drew, 2008; Séguin, 2011). Furthermore, wood has become an important topic in carbon sequestration offsetting (Frank et al., 2010; van der Gaast et al., 2018; Anderegg et al., 2020). Tree ring features are also used to reconstruct past climate (see, e.g., Fritts, 1976; Speer, 2010; Esper et al., 2016, 2018; Ljungqvist et al., 2020a) and for archaeological dating (e.g., Schweingruber, 1988; Baillie, 1995; Ljungqvist et al., 2018). Recently, there is increasing recognition that tree growth, in particular wood formation, is a crucial process for biomass allocation that needs to be explicitly considered in dynamic global vegetation models as part of climate change projections (Fatichi et al., 2014, 2019; Körner, 2015; Friend et al., 2019). As a result of the central importance of wood for forestry, dendroclimatology, dendrochronology, and in fundamental biological research, many models have been constructed to simulate its formation. Nevertheless, there is scope for improving existing wood formation models and to develop new models.

Fritts et al. (1991, p. 114) describe the use of wood formation models as “a beginning effort to serve as an unambiguous medium of communication, which represent the state of knowledge at the present moment as we perceive it.” In this spirit, the history of knowledge increase, hypotheses, and modeling approaches are well-summarized in wood formation models since the 1960s. These have been applied in forestry, dendroclimatology, and the study of wood formation itself. Different mechanisms, environmental or internal drivers of growth, have received attention at various levels of detail. They are a mix of hypotheses on what internally regulates an organism and what physically limits it. Besides a limited, and now outdated, review by Downes et al. (2009), a systematic and process-focused research review on wood formation models has hitherto been lacking. The aim of this review is to summarize the knowledge of growth processes collected in wood formation models, especially with regards to growth–climate relationships and with a focus on carbon. It will highlight some unresolved mechanisms, discipline-specific findings, and the utility and requirements of more data for model-development.

**Figure 1:**
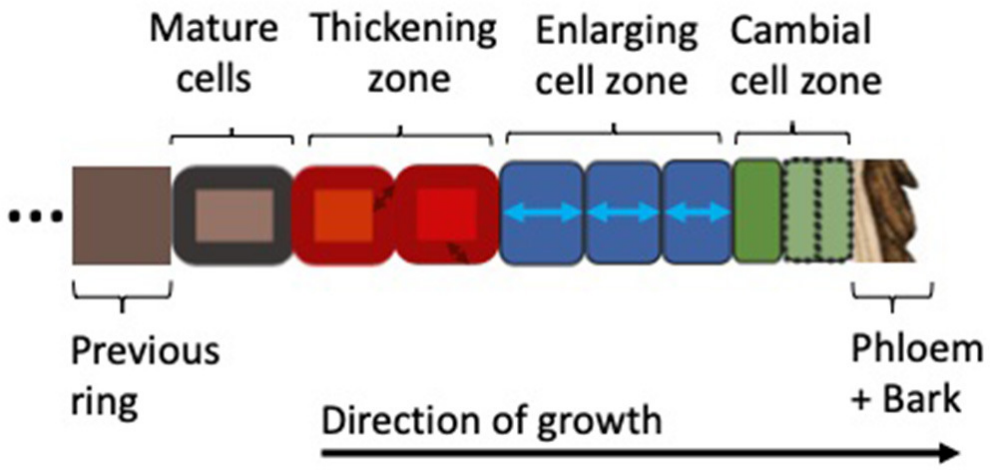
Xylogenesis along a single radial file of developing cells, showing the zones of cell division, enlargement, wall thickening, and mature (dead) cells (Plomion et al., 2001; Fromm, 2013; Rathgeber et al., 2016). Depending on their different stages of development, the cells are assumed to be under varying environmental constraints and tree-internal regulation. Wood formation models covered in this review follow this schema and resolve one or more cell types with their associated processes.

In order to better understand the models reviewed here, we briefly introduce the biological fundamentals of wood formation (i.e., xylogenesis). Xylogenesis involves the production and differentiation of new xylem cells, which eventually mature into functional wood cells (Plomion et al., 2001; Fromm, 2013). Wood formation is a form of plant growth, which can be defined as irreversible expansive and structural growth (Hilty et al., 2021). It follows the same principles as growth in all plants: (1) the production of new cells by stem cells and mother cells in the region called the cambium; (2) the subsequent further radial enlargement of these cells in the enlargement zone; followed by (3) wall thickening, involving the deposition of a secondary cell wall, which in the case of woody plants can be very thick and in addition to cellulose is also lignified to provide extra rigidity and hydrophobic properties; (4) the programmed cell death which transforms mature xylem cells into functional tracheary elements. The wood formation processes are pictured in Figure 1; the biological basis for wood formation is also well-summarized in Rathgeber et al. (2016).

This review covers 17 wood formation models (see Table 1) from the first in 1968 to the most recent in 2020. A brief history is followed by analysis of specific topics such as different mechanistic hypotheses, discipline-specific findings, and data needs. All 17 wood formation models are contrasted based on their levels of complexity and environmental vs. internal drivers/regulators. The baseline for this comparison is the first computer model of wood formation, viz. Wilson and Howard (1968). This model is chosen as the baseline because of its sole focus on cell type-specific processes and its lack of any environmental influences. Thus, any model containing all cell types *and* environmental/tree-internal factors is usually more complex than the baseline model. Models located below or at the same level as the baseline model usually only contain a subset of cell types and processes relevant for wood formation. Exceptions to the latter exist however, and will be described as such.

**Table 1:** Wood formation models covered in this review.

**Reference**	**Discipline**	**Inputs**	**Cell types simulated**	**Aims of the model**	**Species**
Wilson and Howard (1968)	Fundamental research	–	CAM, ENL, THK, MAT	Test a cell developmental framework for secondary growth	*Pinus resinosa, Pinus strobus*
Howard and Wilson (1972)	Fundamental research	–	CAM, ENL, THK, MAT	Test influence of stochasticity on (above model's) rates and transition thresholds	*Pinus resinosa*
Wilson (1973)	Fundamental research	Signalling compound concentration	CAM, ENL, THK, MAT	“Provide new insights into cambial activity”	*Pinus resinosa*
Fritts et al. (1991)	Dendro-climatology	soil moisture, daylength, temperature	ENL	Contribute to understanding of tree ring-climate relationships	*Pinus sylvestris, Pinus ponderosa*
Deleuze and Houllier (1998)	Forestry	temperature, soil moisture, carbohydrates, +)	CAM, ENL, THK	Use a simple model to "understand or simulate the effects of changing environmental conditions [..] on forest production"	*Pinus sylvestris*
Fritts et al. (1999), TreeRing 3	Dendro-climatology	water stress (function of stomatal resistance), carbohydrates, temperature, hormones	CAM, ENL, THK, MAT	"Exactly how do trees record environmental information in the structure of their growth rings in both temperate and tropical environments?"	*Pinus ponderosa*
Vaganov et al. (2006), VS-model	Dendro-climatology	soil moisture, temperature, daylength,	CAM, (ENL, THK)	Construct a model "to achieve wide application to the study of tree ring dynamcis in dendrochronology"	
Drew et al. (2010), CAMBIUM	Forestry	xylem water potential, temperature, carbohydrates, hormones	CAM, ENL, THK, MAT	"[P]rovide a physiologically plausible and testable platform to assist in the understanding of the causes of wood property variation."	*Eucalyptus spp*.
Hölttä et al. (2010)	Fundamental research, forestry	xylem water potential, carbohydrates, temperature, *)	CAM, ENL, THK, MAT	Link cambial growth with tree-level processes such as transpiration and photosynthesis	*Pinus sylvestris*
Drew and Downes (2015)	Forestry, Fundamental research	xylem water potential, temperature, carbohydrates	CAM, ENL, THK, MAT	Provide framework for testing wood formation concepts and highlight areas of research	*Pinus radiata*
Schiestl-Aalto et al. (2015)	Fundamental research	temperature, carbohydrates, prescribed growth curve	CAM, (ENL, THK)	“[P]rovide a framework for [whole-tree] carbon consumption related to cambial growth"	*Pinus sylvestris*
Hartmann et al. (2017), XyDyS	Fundamental research	signalling compound concentration	CAM, ENL	"[Assess] the predictions of the morphogenetic gradient theory."	*Pinus sylvestris*
Cartenì et al. (2018)	Fundamental research	carbohydrates	ENL, THK	Understand the impact of (assumed to be) seasonally increasing carbohydrate availability to the radial file on the "general anatomical pattern of tracheids across the tree ring and the rate and duration of cell enlargement and cell-wall formation"	*Pinus cembra, Picea abies, Larix decidua, Picea mariana*
Hartmann et al. (2021), XyDyS2	Fundamental research	two signaling compounds' concentration	CAM, ENL	"[I]nvestigate the potential of the crosstalk between two biochemical signals in controlling tree radial growth, wood formation, and tree-ring structure"	*Pinus sylvestris*
Friend (2020), RINGS	Carbon studies	temperature, carbohydrates	CAM, ENL, THK, MAT	Investigate 1) “mechanisms for the observed high sensitivity of cell-mass density to temperature within the latewood,” 2) “the influence of carbohydrates on the density profile” 3) “the effect of changing zone widths”	*Pinus sylvestris*
Cabon et al. (2020)	Fundamental research	temperature, water	CAM	"[assess] the biophysical effect of [temperature] and [water potential] on cambial cell enlargement and division"	*Picea abies Larix decidua*

CAM, cambial cells; ENL, enlarging cells; THK, thickening cells; MAT, mature cells. The author's aim of the model is, where explicitly stated, quoted from the publication. All models are run at daily time-steping, unless otherwise highlighted in the “Inputs” column: *) = subdaily, +) = weekly.

Selection criteria for models included in this review are that they simulate one or more xylogenesis processes such as cell division, enlargement or thickening, and the respective cell types at the scale of a radial file (Figure 1). The dynamics should be resolved in a sufficiently mechanistic manner that process-hypotheses can be compared across models. Not considered in this review were models which do not follow the wood formation model framework introduced in Figure 1. These models are most commonly whole-tree models that produce intra-ring features of growth dynamics, often along the whole stem, instead of a single radial file. For example, hormonal flow from the crown received attention by Kramer (2001) in a whole-tree auxin-only model of cambial growth and orientation, where the cambium is approximated as a cylindrical surface along the modeled tree. Modeling of auxin flow along the stem of a tree also plays a large role to determine wood orientation (Kramer, 2002). Other models were developed to simulate timber quality characteristics and do not explicitly consider wood formation, but rather volume and mass increment leading to intra-ring features. These are mostly driven by carbon allocation and water-transport based on the Pipe Theory (Mäkelä and Mäkinen, 2003; Deckmyn et al., 2006). Other models not considered in-depth here can be categorized as radial growth or stem increment models, which do not disentangle increment dynamics into enlarging and cell production components (Steppe et al., 2006; Chan et al., 2016; Mencuccini et al., 2017; Eckes-Shephard et al., 2021; Peters et al., 2021) Overall, most of these models either do not consider the cambium as the driving feature of cell production, or explicit cell enlargement and thickening as the underlying processes for intra-ring patterning and are therefore not considered further in this review.

## 2. A Review of Wood Formation Models

**Figure 2:**
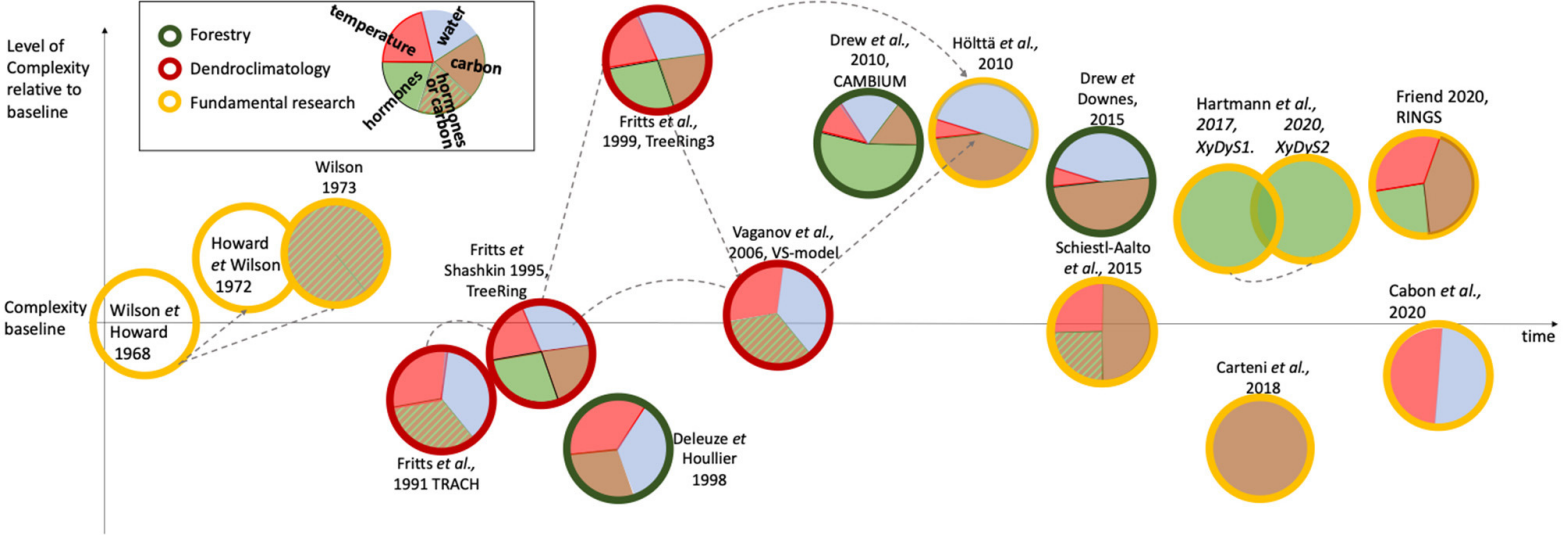
Schematic view of wood formation models over time, their level of complexity, and the environmental/tree-internal influences used within them. Differently colored circles represent the principle focus of the model. Where appropriate, gray dashed arrows highlight structural similarity between models (discussed in more detail in the text). The pie chart proportions represent relative approximate levels of comprehensiveness by which an environmental- or tree-internal- factor (e.g., hormone, carbon) influences model outcome. Striped pie chart components represent a daylength signal, that according to the authors can either be interpreted as carbohydrate availability for growth or a daylength-dependent hormonal signal. Model position on the vertical axis reflects relative complexity. Wilson and Howard (1968) is used as the standard baseline complexity level as, while it considers all cell phases and includes simple transition rules, it does not resolve any environmental or tree-internal regulatory factors. Models below this baseline either contain fewer processes of wood formation e.g., only resolve enlargement and thickening, as in Cartenì et al. (2018), or do not resolve other aspects on wood formation e.g., Deleuze and Houllier (1998) do not resolve a cell undergoing all developmental stages, but assume that it matures within one time step (= 1 week). Some models are integrated into single-tree models (e.g., Fritts et al., 1999; Hölttä et al., 2010; Schiestl-Aalto et al., 2015), but this is not considered in the complexity ranking. Models considered of high complexity either regard all cellular processes and transitions, as well as environmental influences with significant detail (e.g., Fritts et al., 1999), resolve more than one cell type (e.g., Drew et al., 2010), or resolve some processes at such levels of detail that for stability reasons they must run on a very small time step (i.e., 1 s, e.g., Hölttä et al., 2010).

This section introduces the models covered by this review (Table 1) in a chronological fashion. It describes and contrasts models in their complexity and environmental drivers (Figure 2), their applications and the evolution of ideas therein, to provide background to the subsequent sections.

### 2.1. The First Models

The first computer model of wood formation was developed to summarize current knowledge of wood formation processes (Wilson and Howard, 1968). It did not consider environmental impacts on wood formation, shown by the empty circles in Figure 2. Instead, it was concerned with verifying the concept of the wood formation framework (Figure 1). With prescribed rates as input, and very rigid rules for transition between cell types, the model remained of a rather descriptive nature. The first hypotheses which were tested with such models were the impact of stochastic influences on growth parameters (Howard and Wilson, 1972). Howard and Wilson (1972) simulated 16 radial files to study the impact of stochasticity on production, expansion and thickening rates along with zone widths during wood formation on the resulting anatomy of the cells. They find that stochasticity adds too much variability between the files, which makes them conclude that some exogenously imposed signal is necessary for between-file coordination in trees, later to be explained by a hormonal gradient (e.g., Uggla et al., 1996). The then still hypothetical gradients of hormones in the developing radial file were achieved in a model by Wilson (1973) which stimulated hormones that can diffuse across the developing file. Besides slight changes in seasonal hormone input concentration, a “growth sensitivity” parameter was also required to change throughout the growing season in order to obtain a good model–data fit for red pine (*Pinus resinosa*). Wilson (1973) hypothesized that this changing of the parameter could be mechanistically attributed to water availability.

### 2.2. Dendroclimatology Gets Involved

None of these early models explicitly resolved specific environmental influences until dendroclimatology turned to wood formation modeling (Fritts et al., 1991). Dendroclimatology is a discipline primarily concerned with extracting climate information from tree rings (Fritts, 1976; Esper et al., 2018). It follows that their models would naturally resolve what dendroclimatology perceives as the main environmental drivers (or limiters) of growth. Until then, the discipline had exclusively relied on statistical methods to reconstruct climate from tree-growth patterns (Fritts, 1976; Schweingruber, 1988). In using mechanistic modeling of tree growth, dendroclimatologists were attempting to obviate the need to assume linearity and stationarity when studying climate–growth relationships (Tolwinski-Ward et al., 2011; Støve et al., 2012; Ljungqvist et al., 2020b; Wilmking et al., 2020). To address stationarity, it was required that models could vary in the strength of the relationship between growth and an environmental factor. To address linearity, it was required that models accommodate the more biologically realistic non-linear response of growth to environmental factors, for example the decrease in growth activity at very high temperatures (Wilson et al., 2007; D'Arrigo et al., 2008; Ljungqvist et al., 2020b).

TRACH (Fritts et al., 1991) was the first published wood formation model that considers environmental influences, such as temperature, soil moisture, and daylength, to calculate a growth response to the environment. This model is a direct forerunner of the VS-model, now widely used in dendroclimatology. For example, relative growth responses (between 0 and 1) to temperature, water, and daylength are combined to calculate a “common growth response” (in later publications called an “integral growth response”). In TRACH this common growth response can be modeled in two ways, by multiplying all environmental factors (multiplicative model of growth), or as a limited model of growth, where the common growth response reflects the effect of the most limiting factor only (Fritts et al., 1991). The latter approach is adopted in subsequent dendroclimatology models such as the VS-model. The common growth response drives daily changes in cell size from a user-prescribed input of number of cells, combined with either information on mean cell diameter or ring with. As wall thickening is not mechanistically represented, it is derived using an empirical relationship. A degree-day approach is used to initiate cambial activity in the spring in temperate climates. To obtain individual cell sizes within the ring, the integral of the common growth response over the growing season is discretised and an algorithm deployed to transform small intervals of this integral into cell size increment of the prescribed number of cells. The ideological beginnings for this approach are based on the TRACHeidogram technique by Vaganov (1990). Cell-wall thickness in TRACH is then calculated based on the two empirical relationships between cell size and wall thickness as observed by Vaganov (1990). A single radial file is modeled and at the end of the year holds information on cell size, wall thickness, and therefore wood density. The model output was compared with the performance of a statistical model and both were equally capable of reconstructing past ring width (Fritts et al., 1991).

TreeRing (Fritts and Shashkin, 1995), largely developed by Alexander Shashkin, superseded TRACH a few years later. Its representation of the delineation of the cambial zone—first through linear functions (Fritts and Shashkin, 1995), and in a later version through exponential functions (Fritts et al., 1999)—form the basis of the representation of cambial activity in the VS-model (in which this part of the model is called “cambial block”). Besides zone delineation, the two models are conceptually similar in the treatment of cambial activity response to the environment and cell position. This new cambial block enables TreeRing and the VS-model to ultimately simulate tree ring width in response to the environment, not only by enlarging a predefined number of cells (as done in TRACH), but by simulating the cells themselves. In combination with additional inputs such as mean and minimum cell size, a tree ring could be simulated.

The complexity differences between TRACH, the latest version of Treering3, and the VS-model are large (Figure 2). Nevertheless, these models share many concepts related to how environmental influences are evaluated and how these drive cell differentiation (where applicable). For example, all three models combine relative growth responses (between 0 and 1) to temperature, water, and daylength (or carbon availability for TreeRing) to calculate the “common growth response” based on the principle of a limiting factor. This common growth rate, in the case of TRACH, is applied to cell size only. In the VS-model it drives cell production (and separately cell size), and in TreeRing3 impacts cambial, enlargement, and thickening activities in different ways. More specifically, TreeRing3 simulates the rates of all three growth processes based on complicated interactions between regulating factors, such as hormones, a cell's position within the cell development zone, and the integrated growth rate as a function of water, temperature, and carbohydrates. The VS-model is less complex but inherits aspects of the above two models (see also gray arrows in Figure 2).

The VS-model (Vaganov et al., 2006, 2011) and its various derivatives (Tolwinski-Ward et al., 2011; Shishov et al., 2016, 2021; Popkova et al., 2018) have so far been the most applied and published wood formation models in the discipline of dendroclimatology. Not all derivatives (e.g., Tolwinski-Ward et al., 2011; Tychkov et al., 2018) cover the definition of a wood formation model used in this paper. For example, Tychkov et al. (2018) do not resolve any cellular processes, and Tolwinski-Ward et al. (2011) resolve them at a monthly time step. The VS-model's success especially in reconstructing standardized ring width indices in response to the environment has resulted in simpler model spin-offs based on monthly environmental growth rate reconstruction only (VS-lite) (Tolwinski-Ward et al., 2011). The VS-model, along with the cambial block (developed by Alexander Shashkin), is able to simulate cell proliferation in a sophisticated manner. It combines the influence of environmental factors such as water, temperature and daylength as either a proxy for hormones or carbon, in a common relative growth rate similar to Fritts et al. (1991), which modifies cambial growth rates and zone width. The VS-model's most recent cambial zone framework (Vaganov et al., 2011) is based on Treering3, Fritts et al. (1999), which was also used in Hölttä et al. (2010) (Figure 2). As example of application, using the VS-model, Anchukaitis et al. (2006) simulated TRW chronologies in the southeastern United States. First, they calibrate the VS-model to generate the best fit between synthetic TRW and standardised observed TRW. The pattern of the simulated TRW can then be attributed to the environmental impacts which generated the TRW. They discovered that the pattern in the soil moisture-driven environmental growth rate modifier had changed over time. They suggest that the decreased summer precipitation and resulting soil moisture has developed as a new constraint on TRW in that area in the last 60 years. Anchukaitis et al. (2006) predict this constraint to become stronger with projected decreases in summer precipitation.

A new concept of what drives wood growth is implemented in TreeRing (Fritts and Shashkin, 1995) and Treering3 (Fritts et al., 1999). Besides water and temperature, carbon and hormones (in TreeRing3) were important regulating factors for wood formation (the gray literature also contains a manual for TreeRing2000 (Fritts et al., 2000), which is not considered here). Any of these four factors (water, temperature, carbon and hormones) remained as the building block of regulating factors (either as a normalized scalar or explicitly and mechanistically modeled) for all subsequent models (see Figure 2), usually along with the structure of developing and transitioning cells as proposed by Wilson and Howard (1968). Overall, with dendroclimatology becoming involved in wood formation modeling, the link between growth and the environment started to be explored more thoroughly, through the consideration of environmental factors.

### 2.3. Forestry Models

In the 1990s, researchers from a third discipline, forestry, started to publish research output on wood formation modeling, with the view to simulate wood quantity and quality, such as density (Deleuze and Houllier, 1998), vessel frequency (Drew et al., 2010) or later microfibril angle (Drew and Downes, 2015). While dendroclimatology was then mostly concerned with the modeling of simple conifer wood formation processes, forestry also explored the modeling of new species as well as new growth–hypotheses.

The first forestry model by Deleuze and Houllier (1998) is very parsimonious and considers a collection of simple two-parametric equations that transform temperature into a number of cells, soil moisture into volume and carbon availability into mass increase, respectively. So far dendrochronology models such as TRACH, TreeRing3 or the VS-model, had combined all environmental factors to act upon a cellular process e.g., enlargement. This new model differentiates between cell types and their assumed distinct environmental sensitivities to different growth processes (temperature on cell production, soil moisture on cell enlargement and carbohydrate availability on wall thickening). However, another assumption makes the model less biologically realistic: it assumes that a cohort of cells goes through all developmental phases within 1 week. Thus, environmental influences and carbon availability of a single week impact upon volume and mass increase within that batch only. In contrast, when studying observations of xylogenesis, the following picture of cellular dynamics throughout the season is apparent: What is generally observed (Plomion et al., 2001; Fromm, 2013; Rathgeber et al., 2016) is that initial dynamics of xylogenesis during the growing season see the increase of cambial cells first. After a few weeks, some of these cells transition into enlarging cells. Their numbers quickly increase and then slowly decrease throughout the rest of the season. The decrease is accompanied with an increase in thickening cells. Often thickening cells can still be visible while the cambium is thought to be no longer active. This description of the empirical observations shows that the model assumption of all three processes (division, enlarging and thickening) concluding for cell cohorts in only 1 week may lead to the integration of climate and growth factors at the wrong time of the year, potentially affecting the models' predictive skills. Nevertheless, two publications applying the model have been able to show good overlap with observations both qualitatively (Deleuze and Houllier, 1998) and quantitatively (Wilkinson et al., 2015).

Forestry also produced other models which are in their complexity similar to TreeRing3 (see Figure 2). CAMBIUM (Drew et al., 2010) explores hormonal diffusion as a function of crown control and its impact on cell growth rate, developmental phases and differentiation into multiple cell types in *Eucalyptus* xylogenesis. Until CAMBIUM, all previous models had been developed on softwoods, which have simple cell types (thus easier to model), and of which some grow close to the climatic limits of their distribution (relevant for dendroclimatologists). As CAMBIUM focuses on conceptual morphogenic gradients, environmental factors are represented in less detail, through an environmental modifier which includes the influence of water and temperature, in a manner similar to dendroclimatology models, based on the principle of a limiting-factor. Carbohydrate availability also influences cambial, enlargement and thickening activity. The CAMBIUM model heavily invoked an interpretation of the canalization hypothesis (Sachs and Cohen, 1982) and radial auxin distribution findings of Uggla et al. (1996) for its cell fate determination algorithm.

An example demonstrated on two forestry models is some models' structure-dependent, intrinsic reliance on specific environmental factors to obtain a desired feature in the tree ring. Annual tree rings are common to trees in temperate zones, as is a distinct increase in wood density within the ring toward the end of the season. The different regions of low and high density are called earlywood and latewood, respectively. In middle and high latitudes early and latewood commonly form early or later, respectively, during the growing season. Drew and Downes (2015) as well as Deleuze and Houllier (1998) developed forestry models to simulate and study wood property variations, especially wood density. Both models use very different modeling approaches, but both simulate density reasonably well, with Deleuze and Houllier (1998) being able to recreate the relative patterns, and the more complex model by Drew and Downes (2015) being able to replicate up to 80% of the variation within the mean sample wood density observations in tree rings. However, for density to sufficiently increase toward the end of the year, Deleuze and Houllier (1998) rely on climatic conditions to be dry. At mesic sites, the model will not decrease its volume increment, as this process is directly and uniquely dependent on water availability. Similarly in Drew and Downes (2015) latewood is induced by soil moisture stress, but in addition, latewood is induced by a switch in the model, which is related to the day of the year. In the Australian context in which it was developed and applied, the model was used to explore potential wood density shifts in pine plantations under future scenarios in which water availability varied and temperature increased (Drew et al., 2017).

#### 2.3.1. Physiological Models of Wood Formation in Forestry and Fundamental Research

Until the 2000s, most wood formation models were not of a physiological nature. What this means is that growth or wall thickening rates were largely determined based on a combination of scaled relative growth rates. These follow general response-function type relationships. Physiological models of wood formation are concerned with biophysical and biochemical mechanisms that result in growth dynamics within and between cells in response to environmental conditions. Specifically, these models consider the mechanisms that underlie cell proliferation, enlargement or wall thickening processes. For example, they may resolve the interaction between hormonal concentrations on a given day and their hypothesized influences on cell wall elasticity, from which an enlargement rate emerges (Drew et al., 2010). Likewise, the thickening rate may emerge out of a combination of carbon availability, based on the position of the cell in the developing radial file of the tree ring, and temperature (Friend, 2020). Increased computational efficiency made it possible for processes to be resolved and studied at such levels of detail. This trend is also shown in Figure 2, where models become increasingly complex. Yet some models stand out from this trend. These models either only mechanistically resolve a subset of cell types and processes (e.g., Fritts et al., 1991; Vaganov et al., 2006; Cartenì et al., 2018; Cabon et al., 2020) or do not consider transition between cell types (Deleuze and Houllier, 1998). The lower complexity may be the result of various reasons. For example, these models may not require a higher level of detail for their research questions, e.g., Vaganov et al. (2006) simulate only tree-ring width (TRW) for climate reconstruction purposes.

Physiological models are able to explore hypotheses on certain drivers (e.g., water or carbon), regulators (e.g., hormones) or processes (e.g., thickening) at high levels of physiological detail. For example, Hölttä et al. (2010) published a complex model calibrated to Scots pine (*Pinus sylvestris*) that explicitly treated water diffusion through the stem and individually modeled cells. Sugar transport was modeled based on diffusion; a cell's water potential was based on water and sugar content, of which both entities diffuse through the developing xylem. Growth in cambial and enlarging cells was turgor-driven, cell division based on a size-threshold value, and cell wall synthesis rate based on sugar content. Diffusion of water across the developing cells in the file required very small (<1 s) time-stepping to remain stable. Another forestry model by Drew and Downes (2015) explores water and sugar interactions on enlargement, this time in Monterey pine (*Pinus radiata*) using a different, optimisation-based approach, where sugar is considered the primary driver of osmotic potential and therefore turgor. This places the model marginally below (Hölttä et al., 2010) in terms of its complexity (Figure 2). The influence of hormonal control was handled differently compared to CAMBIUM (Drew et al., 2010), with a focus in the 2015 model on the influence of turgor on cell expansion. Specifically, Drew and Downes (2015) assume a 3D (rectangular prism) cell and use an optimization routine to determine how many cells were able to expand given each cell's volume and the amount of available sugar and estimated water deficit. Drew and Downes (2015) also explore a novel approach with regards to cell wall thickening, which besides carbon availability is dependent on the dynamically-changing cell lumen surface area.

### 2.4. From Direct Applications to Fundamental Research and Hypothesis Testing

While many of the forestry models discussed above also had fundamental research in mind, their dominant aim can be considered to be practical applicability in forestry. Recently, numerous models intended for fundamental research have been built with the exclusive aim to test different hypotheses, increase our knowledge on wood formation processes, explain open questions or challenge existing ideas. The latest models have largely taken up the idea of hormonal regulation at various levels of detail. A morphogen-only model (XyDyS) was developed by Hartmann et al. (2017) and extended (XyDyS2) in 2020. Hartmann et al. (2017) and Hartmann et al. (2021) simulate the explicit diffusion of a morphogen (such as auxin), and an additional compound (Hartmann et al., 2021) and found that two interacting compounds, acting as morphogen and process rate-determinants, are needed to explain the seasonal kinetics of cell differentiation and final tree-ring structure. The two models are in aim and approach very similar to the first ever hormonal model (Wilson, 1973), but include much more reference to recent molecular knowledge such as protein-channel mediated diffusion of auxin.

All models have, until recently, considered cell enlargement and wall thickening as two separate processes. Cartenì et al. (2018) challenged this idea and could indeed replicate the patterns observed in tree-ring density profiles when combining these two processes. In order to focus on the enlargement-thickening processes, they omit cell production (in a similar manner to TRACH). The consequence, and one of their core model assumptions, is the need for an increase in carbon supply to wood formation toward the end of the season to replicate an increase in density in the latewood sections. In contrast, a constant amount of carbon allocated to the developing cells, but with decreased cell production toward the end of the season reproduces realistic tree ring density profiles in RINGS (Friend, 2020): RINGS incrementally decreases the zone widths of the developing cell types toward the end of the season. This increases the amount of carbon available to late forming cells. With this zone-width approach the model avoids the explicit modeling of e.g., a hormonal signal across the radial file. RINGS is able to reproduce both intra-seasonal cellular dynamics and final ring density patterns well. Friend (2020) could also use the zone-width patterning to explain compensating effects of growth dynamics in response to environmental factors observed by Cuny et al. (2019). Other hypothesized mechanisms related to wall thickening that result in latewood formation will be discussed in another section below.

**Figure 3:**
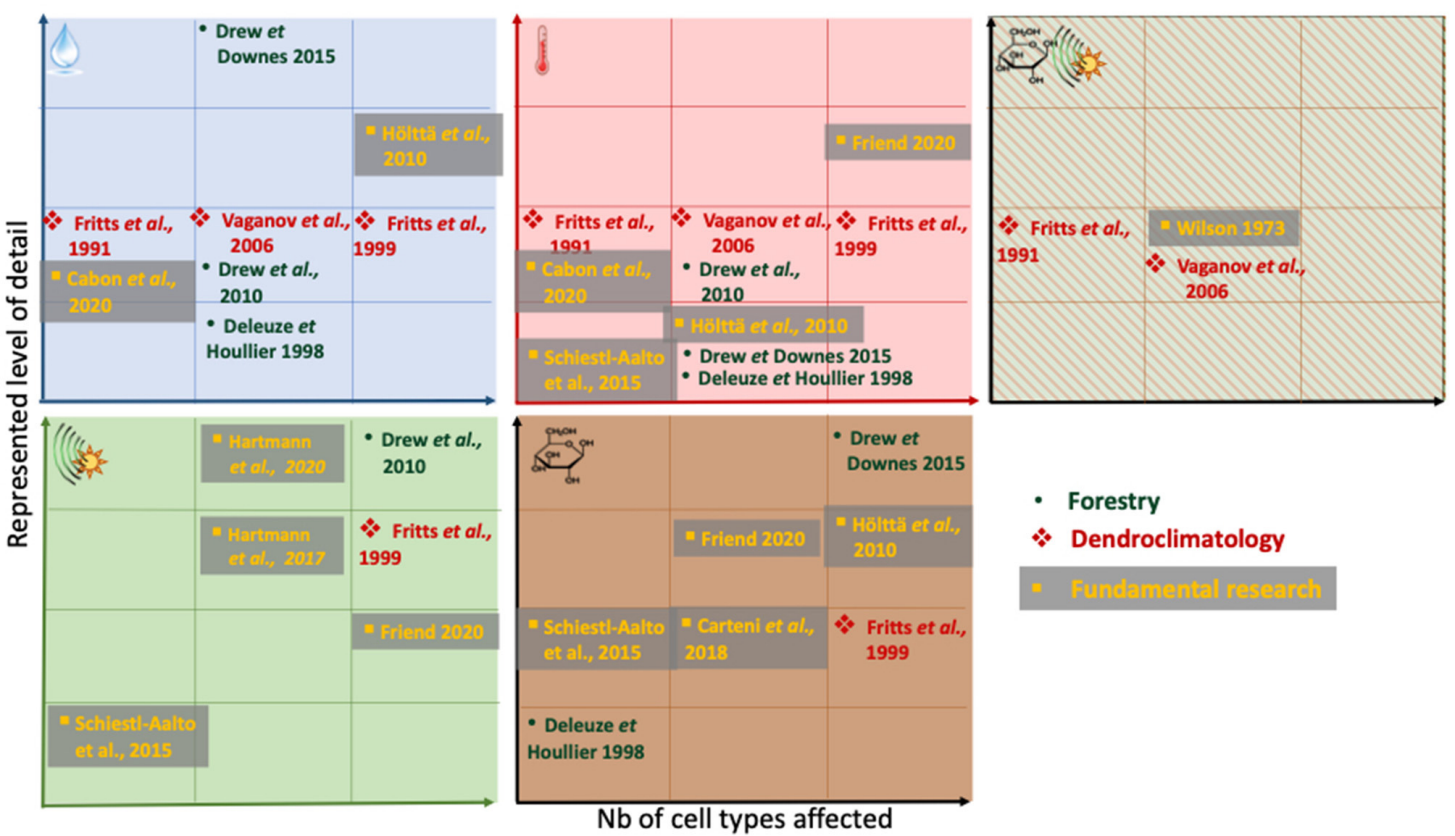
Schematic of environmental and tree-internal drivers and regulators represented in the wood formation models discussed in this paper. Drivers reported are (top-left to bottom-right) water, temperature, either daylength (phenological) signal or carbon, hormonal/daylength (phenological) signal, carbon (note the absence of nutrients as growth rate modifiers in all models). The positioning along the axes within each box reflects (1) the number of cell types affected by an external/internal driver and (2) the level of detail driver-cell interactions are resolved. Left to right: First square: one cell type is affected only (this could be e.g., cambial cells or wall thickening cells only), last square: all three cell types are affected. Bottom to top: low level of complexity with which an environmental driver influences the model e.g., as single part of a physical equation (e.g., through a threshold parameter to promote an on-off switch environmental switch (e.g., assume metabolic activity occurs only above 5°C (Deleuze and Houllier, 1998))) next square: as part of a response-function e.g., increasing enlargement rate with temperature (Fritts et al., 1991; Friend, 2020). Complexity can increase even further to a detailed physiological level, for example through spatial interactions e.g., diffusion of carbon across the developing radial file, followed by arriving carbon being included into the thickening cell wall, as sort of done in Friend (2020). Note that for Schiestl-Aalto et al. (2015), we interpret the “ontogenetic development” used as one the modifiers of cell proliferation dynamics by the authors, as an internal signal, and therefore place it in the bottom-left square, as a hormonal/daylength (phenological) signal. If a model is absent from an environmental factor matrix, it does not resolve this particular environmental factor. Within the squares, no ranking is attempted. Note that the relative position along the y-axis is not comparable between the environmental /internal factors. Note that this is a rough scheme, derived from text-descriptions and equations in the publications, which sometimes may not represent the entirety or complexity of an operation in the model itself. Only the latest version of Treerings (Treerings3) is shown here.

The timings and significance of individual environmental and internal drivers on tree growth continue to be unresolved and therefore recent models still work on addressing these seemingly fundamental questions. Cabon et al. (2020) describe a cell production model, where a constant number of cambial cells grow in size dependent on water (turgor) and temperature. Division at a threshold value leads to one cell immediately leaving the cambial zone. The model is tested against the final number of cells at the end of the year and within-season cell production dynamics. Keeping some environmental drivers constant in different simulation scenarios, they find that the model required variable temperatures to explain tracheid production onset, and that water potential, probably even trunk water potential, may be necessary to better simulate production cessation and number of cells produced. To investigate other fundamental but unresolved questions related to intra-tree carbon source-sink dynamics and the environment, Schiestl-Aalto et al. (2015) use a wood formation component in the whole-tree carbon-balance model CASSIA. Therein, when growth is active, cambial cell numbers are determined by 1) temperature, 2) an empirical term reflecting commonly-observed patterns of intra-seasonal cambial activity levels (referred to as "ontogenetic development"), 3) carbon availability from storage, and 4) photosynthetic activity (Figure 3). Enlarging and thickening cell numbers on a given day depend on the duration spent in their respective stage of development, which is 1) driven by cambial cell production and 2) earlywood and latewood fraction of the calibration year. The model-focus is not on physiological details and individual cells and their dynamics are not explicitly considered. Therefore, it is in its structure one of the more parsimonious models covered here. However, due to the environmental and internal drivers on cell production, it is placed at a similar level of complexity as the baseline-model (Figure 2). The authors use this framework within a whole-tree model to determine the impact of environmental factors on growth (sink) activities in cold environments. They found that stored carbon did not limit intra-annual growth, whereas temperature did.

Knowledge increase through fundamental research was also the aim of the very first wood formation models. We have gone full circle across more than half a century of wood formation modeling since the 1960s. The discipline-specific wood formation models have already helped answer a wide-ranging suite of questions, from improving our knowledge on fundamental growth hypotheses, to wood quality prediction and attributing large-scale climatic impacts to observed growth patterns. The next section will summarize and discuss old and new model hypotheses for various selected mechanisms, in context with new and old observations. This includes open questions about growth mechanisms. Furthermore, data needs, new software and new areas for wood formation modeling are discussed.

### 2.5. Current Knowledge, Open Questions and Future Opportunities

The historic overview of wood formation models contrasted the models in terms of their level of complexity relative to the baseline model by Wilson and Howard (1968) (Figure 2). It further highlighted the diversity in modeling approaches over time, and pointed out the breadth in wood formation model applications and findings (from the cellular to the regional). This section will examine how unresolved process are modeled (specifically: hormones, earlywood–latewood transition and the involvement and representation of sugars in different cell developmental phases), discusses existing and novel data useful for model parameterisation and testing, and finally turns to additional disciplines where wood formation modeling is useful but still in its infancy, such as in carbon storage modeling and global vegetation modeling.

#### 2.5.1. Wood Formation Process Hypotheses: Resolving Hormones

Hormones have been hypothesized to play a key role in determining aspects of wood formation since at least Larson (1960). While many hormones are thought to be involved (reviewed by Buttò et al., 2020a), most wood formation models today explicitly treat one (Fritts et al., 1999; Drew et al., 2010), at most two hormones (Hartmann et al., 2021), with good model–data fit (but see Hartmann et al., 2017). This section reviews the different model strategies to represent auxin, the hormone that is most commonly referenced within the wood formation frameworks.

New observations have both enabled the testing of new hypotheses, as well as acted as additional source to compare models against. Models have also suggested hypotheses before the emergence of data in support of it. For example, Wilson (1973) assumed in his hormonal diffusion model that regulatory compounds (hormones) must be entering the developing radial file from the phloem, then diffusing radially inward, thus creating a concentration gradient across the developing file. While evidence from tissue culture (Wetmore and Rier, 1963) at this time was already strongly suggestive of such hypothesized gradients (Wilson and Wilson, 1961), methods were still insufficient to directly measure a concentration gradient across the first 2 mm of the phloem or developing xylem. In simulating a compound diffusing through the developing file and interacting with a second potential compound, Wilson could reproduce the cell radial diameters of a red pine (*Pinus resinosa*) annual ring grown during a year with summer drought. This modeling exercise added to the emerging evidence of compound-diffusion across the tissue. A “steep radial concentration gradient” of auxin was indeed found 23 years later in Scots pine (*Pinus sylvestris*) by Uggla et al. (1996), followed by hybrid aspen (*Populus tremula L. x Populus tremuloides Michx*) (Tuominen et al., 1997), and was hypothesized to be involved in regulating cell identity (Uggla et al., 1996) and growth-differentiation rate (Aloni and Zimmermann, 1983; Tuominen et al., 1997).

According to observations, a hormone such as auxin seems to be actively involved in regulating cell enlargement rate under non-limiting conditions (Du et al., 2020) as modeled in Wilson (1973), Fritts et al. (1999), Drew et al. (2010), and Hartmann et al. (2021), but not in Hartmann et al. (2017). It is observed to act as a positional signal for cell identity (Uggla et al., 1996; Bhalerao and Fischer, 2014), as modeled in Wilson (1973), Fritts et al. (1999), Drew et al. (2010), Hartmann et al. (2017), Hartmann et al. (2021), Friend (2020), and Vaganov et al. (2006) for cambial cells. Vaganov et al. (2006) is presently the only model in which cambial zone width can change with environmental conditions. The above seems to suggest that models contain robust hypotheses when it comes to auxin-related processes. However, while these models assume that auxin works in a dose-dependent manner, no specific concentration threshold has yet been identified that can delineate zone widths, or no auxin-concentration dependent growth-rates have been measured, two fundamental assumptions of most of these models.

Moreover, the existing observations and models are inconclusive as to whether the morphogen (auxin) is also directly required for growth rate regulation (e.g., Friend (2020); Hartmann et al. (2021) assume no influence on growth rate by auxin whereas Wilson (1973); Fritts et al. (1999); Drew et al. (2010) do assume auxin modification on the growth rate). Some models and observations seem to suggest that spatial (length of the developing zones) interactions with tree-internal factors (morphogens) go hand in hand (Uggla et al., 1996; Tuominen et al., 1997; Friend, 2020; Hartmann et al., 2021). On top of that, environmental (e.g., end of season drought or lower temperatures) factors influence either (1) the size of the zone (Vaganov et al., 2006) or (2) growth rates directly (Wilson, 1973; Drew et al., 2010; Friend, 2020).

All hypotheses used in the models rely on empirical evidence upon which to base their assumptions. The plethora of model approaches with which anatomic (sometimes together with dynamic) patterns can be replicated, shows that this complex system has many tree-internal and external components, which can regulate the outcome. Wood formation models have helped to formalize hypotheses on hormonal influence and hormonal-environmental interactions in various ways. No approaches can be dismissed outright, as they all replicate observations within the context of their studies. To clarify the current incompatible hypotheses among models, more observations on the interactions between hormones, the environment and wood formation at the molecular level are urgently needed (e.g., Uggla et al., 1996).

#### 2.5.2. Wood Formation Process Hypotheses: Earlywood to Latewood Transition

A currently-relevant and contested question is the mechanism behind the earlywood–latewood transition in temperate forest conifers. The subject remains open to the extent that it is even unclear whether the change in density across the ring is (H1) an emergent property caused by physical limitations to growth, (H2) caused by seasonal changes in carbon availability to the developing tree ring or, or (H3) is caused by the temperate tree's strategy to anticipate future environmental limitations (i.e., winter). The number of hypotheses raised here reflect the number of ways this mechanism is represented in wood formation models.

##### 2.5.2.1. H1: Environmental Limitation Leads to EW–LW Transition

An earlywood–latewood pattern is altogether absent in some low and mid-latitude regions or in diffuse porous angiosperms. For example, conifers growing at low latitudes, where temperature, water availability and daylength are relatively stable, such as in tropical rainforests, do not show an annual distinction between large thin-walled cells and narrow thick-walled cells. Hence seasonal tree rings are hard to discern under these non-limiting conditions since the cambium remains active throughout the year. For example, de Mil (2018) found that many tropical forest trees did not have easily detectable, and only “non-periodic" rings. However, in areas where drought periods frequently occur, such the Bolivian Amazon region, narrow wood cells are formed periodically, and these resulting tree rings can be attributed to precipitation (Brienen and Zuidema, 2005). Similar responses to rain and dry seasons have been observed in teak (*Tectona grandis*) wood in Ivory Coast of West Africa (Dié et al., 2012). Tree rings in the tropics also form under conditions of flooding, when the roots do not receive enough oxygen, temporarily arresting growth (Worbes, 1985, 1995). These empirical evidences indicate that environmental limitations such as drought and wet seasons can at least cause patterns similar to high and mid-latitude earlywood and latewood.

That earlywood–latewood transitioning is a consequence of environmental (water) limitation (Hypothesis 1) is covered by the DH-model (Deleuze and Houllier, 1998). In their model they assume that cell enlargement decreases under water stress. Similarly, water-related mechanisms, modeled at higher physiological detail, would decrease cell diameter in Hölttä et al. (2010) and Drew and Downes (2015). The latter two models could also be to some degree influenced by an increase in carbon availability (Hypothesis 2). However, in both models carbon increase will not be able to rescue cell enlargement indefinitely. Therefore, Drew and Downes (2015) additionally assume a daylength signal (Hypothesis 3)—dependent decrease in cell size and increase in carbohydrates allocated to individual secondary wall thickening cells. Thus even if no water stress occurs, the desired earlywood—latewood pattern will emerge.

##### 2.5.2.2. H2: Carbon Availability Influences EW–LW Transition

One model exclusively relying on a change in carbon availability to the developing cells toward the end of the growing season is Cartenì et al. (2018). They assume that the thin-walled earlywood cells, followed by thick-walled latewood cells, result from the carbon allocation pattern during the growing season. Specifically, they assume that allocation of carbohydrates to wood formation processes increases when primary growth ends (Cartenì et al., 2018). Additionally, they suggest an alternative approach for the succession between the phase of enlargement to thickening: Until Cartenì et al. (2018), all frameworks had assumed that cell enlargement and wall thickening are two separate processes following Wilson and Howard (1968). The model by Cartenì et al. (2018) tested the hypothesis that these two processes could occur simultaneously and that thickening is the mechanism by which the end of enlargement is determined. In contrast, in their alternative approach, Cartenì et al. (2018) simulate cell enlargement and secondary wall deposition occurring simultaneously within a cell. Enlargement stops once a cell wall grows too thick to further expand. This process-representation is dependent on carbon influx increasing toward the end of the growing season, in order to reach a critical wall thickness sooner and thus obtain smaller cells later during the season.

Defoliation and daylength experiments are cited as the basis for separating these two processes. Particularly, Larson (1964a) finds that modification of hormones through partial crown coverage leads to cells remaining large but having thick walls. Additional direct evidence comes from Larson (1960), who applied auxin to decapitated seedlings within the latewood formation season and induced larger earlywood-type cells. That auxin levels regulate xylem cell size and differentiation is also found in Tuominen et al. (1997). Molecular mechanisms (“acid-growth theory”) for auxin-mediated cell enlargement have been suggested decades ago but had not been validated. According to the acid-growth theory auxin influences ATP-ase activity and thus cell vacuole (and thus cell) enlargement. In recent years strong genetic and biochemical evidence in support of this theory have emerged (reviewed in Du et al., 2020, see also Perrot-Rechenmann, 2010).

Nevertheless, some overlap between cell enlargement and thickening processes has been observed, at least in European aspen (*Populus tremula*). Sundell et al. (2017) found that tissue that was visually determined to be the beginning of the thickening zone had a stronger molecular signature of still being enlarging cells. This means that early thickening cells were either still enlarging or had not yet stopped expressing the genes necessary for cell enlargement. If the former is true, to bring this in context with the hypothesis by Cartenì et al. (2018), there seems to be a small spatial overlap where thickening and enlarging is ongoing simultaneously. However, this area of shared activity within the radial file is relatively narrow and does not indicate that these processes compete to determine cell radial diameter. Instead, with the early indirect evidence of hormonal influences, as well as the increased evidence on auxin-mediated acid-growth theory on cell enlargement, one can tentatively conclude that what regulates cell size under non-limiting conditions is not wall thickening (= carbon), but hormonal signals. Carbon and hormonal manipulation studies will be useful to solidify this evidence.

Many other models also assume the earlywood–latewood pattern to be carbon-related. While some models directly impose carbon-related mechanisms for the transition, other models find that the pattern, though carbon related, does not have to be imposed, but is an emergent property of the late season growth dynamics. The increase in carbon availability, by prioritizing carbon allocation to thickening cells, is a mechanism to ultimately obtain thicker cell walls in Drew and Downes (2015). This is mediated through a daylength-induced hormonal signal, as stated above. Increases in carbon availability to thickening cells emerge naturally through a shorter radial file, where fewer cells are closer to the phloem and share the incoming amount of carbon in RINGS (Friend, 2020). When it comes to spatial representation of carbon across the radial file, interestingly, both Friend (2020) and Drew and Downes (2015) use carbohydrate gradient observations to justify their carbohydrate allocation schemes, with very different effects. Uggla (2001) observe high levels of carbohydrates at the phloem, which then gradually decreases across the developing radial file. Drew and Downes (2015) treat the gradient as an emerging property which is only observable because thickening cells (1) take precedent when it comes to carbon allocation to the cells and these thickening cells then (2) take out more carbon than enlarging cells, thus there are lower nonstructural carbon levels observed in the thickening zone of the radial file. In contrast, RINGS (Friend, 2020) is based on the assumption of equilibrium between diffusion and consumption. In practice this means that RINGS calculates the carbohydrate diffusion profile into the radial file as an outcome of (1) the carbohydrate input into the radial file, (2) each cell's individual demand, and (3) the diffusion itself. More studies exploring not only the seasonal carbon dynamics, but also their gradient across the developing tree ring (as done in Uggla, 2001), would be useful to better deduce the relationship between carbon dynamics and latewood formation.

##### 2.5.2.3. H3: EW–LW Transition as Strategy to Anticipate Future Environmental Limitations

Other models assume that a hormonal signal induces latewood-formation in temperate regions, in line with Hypothesis 3. For example, toward the end of the growing season, a signal from the crown helps to create narrow latewood cells by decreasing enlargement rate in TreeRing3 (Fritts et al., 1999), decreasing zone width and enlargement rates in CAMBIUM (Drew et al., 2010), or by decreasing zone widths and hence enlargement duration in RINGS (Friend, 2020). Empirical studies support these model assumptions by finding that the seasonal growth and development of foliar organs release (auxin) signals, which decline in strength toward the end of the season (e.g., Larson, 1964b). Xylogenesis studies have also correlated the highest cellular activity with daylength (Rossi et al., 2006b; Cuny et al., 2014). In temperate regions, one could expect a daylength-driven signal for trees to anticipate temperature changes, that make growth unfavorable (Petterle et al., 2013). This may be needed in order for the tree not to be surprised by cold temperatures, which may damage immature cells (Rathgeber et al., 2016). Specifically, some processes have to be concluded before unfavorable conditions emerge. For example, lignification is strongly constrained by temperature (Gindl et al., 2000; Körner et al., 2019), and it has been shown that the last xylem cells need up to 2 months until reaching maturity (Cuny and Rathgeber, 2016). In order for these processes to fall into the growing season, the tree can most reliably use daylength as a measure of time progression.

All in all, there seem to be multiple mechanisms which could lead to “earlywood–latewood” patterns and thus tree rings. Some mechanisms are of a physical nature such as water stress in areas not constrained by temperature and daylength, such as tropical regions. Nevertheless, in the temperate regions all maturation processes must be concluded before too low temperatures occur in order to avoid damage. Thus trees might use daylength-perceiving hormones to ‘look ahead‘. Both such mechanisms are implemented in different models. For example, RINGS (Friend, 2020) functions on the hypothesis of daylength-induced zone-width decreases (which are possibly hormone induced). However, RINGS does not contain any water-driven enlargement or stress-function and therefore may not be able to replicate large and small cells at low-latitude sites with water stress. On the other hand, models which rely on water stress only for this pattern to emerge may not work at high-latitude mesic sites (e.g., Deleuze and Houllier, 1998). Fritts et al. (1999) and Drew and Downes (2015) can accommodate for both these conditions in their models. A universally applicable wood formation framework would have to accommodate both physical and tree-internal regulatory mechanisms to replicate intra-annual changes in cell diameters across all latitudes and environmental gradients.

#### 2.5.3. Carbon Availability and Growth Dynamics in Wood Formation Models

Whether growth is actively demanding carbohydrates or passively receiving carbon as a function of photosynthesis is a point of contention (Sala et al., 2012; Dietze et al., 2014), with potential implications on modeling tree growth behavior and ecosystem carbon storage (Leuzinger et al., 2013). Specifically, an open question remains to what degree growth is limited by carbon supply rate (and hence the carbon source) or by its own environmentally and internally-determined activity (and hence itself as the carbon sink). While carbon is necessary for structural and metabolic purposes during xylogenesis, an experimental study by Sundberg et al. (1991) suggests that it is cambial activity, and not carbohydrate availability that determines wood production. Observed NSC concentrations in the cambial region (xylem and phloem) (Giovannelli et al., 2011) can be statistically or qualitatively related to the number of total living (cambial, enlarging and thickening) cells (Deslauriers et al., 2016), and wall thickening or predominantly radial growth periods (Simard et al., 2013). Nevertheless, the direct mechanism by which carbon influences each cell type remains unclear. Therefore, the question remains whether carbohydrate gradients observed across the developing xylem (Uggla, 2001) are, similar to co-occuring auxin gradients, “instructive or incidental” (Bhalerao and Fischer, 2014). While there is some molecular evidence for different sugars acting as signaling molecules for different cellular stages (Riou-Khamlichi et al., 2000), the nature of this relationship has not yet been described usefully for wood formation modeling. How do wood formation models resolve carbon–growth interactions?

The first model that considered carbon explicitly was Deleuze and Houllier (1998), where wall thickening is assumed to be carbohydrate-dependent. This means that in their model, all mass gain of the radial file (through cell wall thickening) is directly related to carbon availability. The first model that considered the influence of carbon on wood growth rates was TreeRings (Fritts and Shashkin, 1995). There, the cell production rate is a function of three limiting factors *F*(*s, W, T*), with water availability *W*, temperature *T* and carbohydrate availability *s*. In a subsequent version, TreeRings3 (Fritts et al., 1999), *F*(*s, W, T*) is involved in deriving the rates of all processes for all cell types. If there was little carbon available, *s* would dominate the equation, if *W* and *T* were not limiting at the time. However, if it was particularly cold or dry, the other factors were able to override this “source dependent” behavior of the model. Indirect growth-rate dependent representation of carbohydrate influence as above is represented in a similar manner in Fritts et al. (1991) and Vaganov et al. (2006). They use a daylength growth-modifier (*F*(*D, W, T*), width *D* as daylength) to influence cell enlargement (or optionally also wall thickening in Vaganov et al., 2006). Both articles say that daylength can either represent a phenological signal from the crown or an indirect representation of photosynthesis and thus carbohydrate availability. In summary, early models already assumed a carbon-dependency of one or more processes, either explicitly as substrate or as rate modifier.

Carbohydrate influences are represented in more complex, physiological ways in recent models. Related to cell production, a cell in CAMBIUM Drew et al. (2010) and Drew and Downes (2015) can only divide if a minimum quantity of carbohydrate is available for this process, giving the model a source-centric behavior. Nevertheless, Drew et al. (2010) and Drew and Downes (2015) also enable storage of surplus carbon in the radial file, which decouples cambial and mass growth, and photosynthesis to some degree, should the environment be favorable to growth again (see also Drew et al., 2009). Secondary wall thickening in CAMBIUM (Drew et al., 2010) is dependent on carbohydrate as substrate, similarly to Deleuze and Houllier (1998).

Besides as substrate, carbon has also been assumed to be a driver in processes such as cell enlargement. For example, Hölttä et al. (2010) and Drew and Downes (2015) explore the influence of carbohydrates together with water availability to represent turgor-driven cell enlargement, making these models also subject to carbohydrate control. As cell enlargement also occurs in cambial cells before division, cell production rates are also carbon-dependent in these models, but due to the small size threshold after which they divide, a carbon-dependent “rescue” of cell production dynamics are probably most prevalent under water-limiting conditions. Both models subsequently use the carbon that contributed to cell expansion as substrate for cell wall synthesis. A constant carbohydrate influx in RINGS Friend (2020) is in contrast with Cartenì et al. (2018), who must assume an increase in carbohydrate supply toward the end of a season in order to obtain an increase in wood density, making the model strongly dependent on 1) carbon availability itself but also, 2) the hypothesized timings of carbon availability to growth. In Drew and Downes (2015), allocation to thickening cells is actively prioritized toward the end of the season, equivalent to an enforced sink demand by wall thickening-cells. In RINGS the density increase toward the end of the ring is governed by increased proximity of the thickening cells to the phloem, the source of carbohydrates. Thus, under normal conditions, according to this model, the only conditions under which carbohydrates are limiting are at the periphery of the developing radial file early during the growing season. This carbon limitation is not however directly caused by the carbon input into the file, but by the physical position of a cell and the diffusibility of carbohydrates across the file. Nevertheless, carbon (source) limitation could influence wall thickening in RINGS, if carbon levels in the phloem are low. Overall, carbon in the above wood formation models is not only assumed to be required as structural component in cell walls of newly formed or wall thickening cells, but also regulates cell enlargement activity by contributing to cell turgor. Some models are more sensitive to intra-annual fluctuations in carbon availability than others. It becomes clear that there are many models which could be subject to source-limitation in one or more of their processes if carbohydrates became limiting and thus the question remains, whether carbohydrates are ever limiting to any of these processes in reality.

Carbon storage regulates cell proliferation in CASSIA (Schiestl-Aalto et al., 2015) by asymptotically declining growth rates dependent on carbohydrate availability after carbohydrate availability falls below a threshold. Using a threshold-only evaluation, Drew and Downes (2015) also have such a safety-mechanism where cell proliferation stops immediately if insufficient carbon is available to build new cell wall plates between dividing cells. Cell cycle studies confirm the plausibility of this mechanism. For example, Riou-Khamlichi et al. (2000) found that carbohydrates act directly as signaling molecules in the cell cycle regulation of *Arabidopsis*. From a tree's perspective, regulating growth at its first process (cambial activity) makes sense as carbon used in wood formation is irretrievable and must be closely regulated to avoid wastage (McCahill and Hazen, 2019). Some evidence suggests that in cases such as under strongly carbon-limited condition, storage is prioritized over growth (Hartmann et al., 2015; Weber et al., 2018), a behavior which may however also strongly be linked to ecological strategy (Mitchell et al., 2016). The concept of a carbon storage threshold limiting growth activity is also a useful framework to connect storage to growth dynamics in models, while still allowing for assumptions on the sink dynamics to remain relatively autonomous from the source otherwise. However, large difficulties will remain to parameterise such a threshold as, if it exists, it may be tissue-, species-, age/size and/or growth environment dependent.

This section has examined the cell developmental processes at which current wood formation models require carbon in order to execute growth dynamics (i.e., irreversible volume or mass growth). With many processes requiring carbon for structural or procedural purposes (metabolism has not been mentioned here), on the wood formation model level, carbon limitation on growth cannot be excluded. Under a low tree carbon status, the source *vs*. sink balance may shift to a sink *vs*. storage story. Under high tree carbon status, wood formation may be limited by environmental factors, while processes requiring carbon are not limited by it. For example, through observations in oak (*Quercus*) (Lempereur et al., 2015) and modeling of larch (*Larix*) and pine (*Pinus*) (Eckes-Shephard et al., 2021), it has been shown that under water-limiting conditions tree growth stops earlier than photosynthesis. Further, modeling of an individual pine (*Pinus*) (Schiestl-Aalto et al., 2019) showed that wood growth variations could be explained by temperature-driven sink activity on a daily basis and that carbon does not seem to be the ultimate driver or limiter of the growth dynamics on a daily timescale. Wood formation models will not in themselves be able to answer the source-sink controversy, especially with the added complexity of storage competing with growth under some conditions. Nevertheless, wood formation modeling can play a useful role to better study the interplay between photosynthesis, storage and biomass increase. With the exception of Schiestl-Aalto et al. (2019), there seem to be no modeling studies explicitly resolving wood formation to interrogate how source-sink-storage relations interact on an intra-annual scale.

### 2.6. Model–Data Comparison

**Figure 4:**
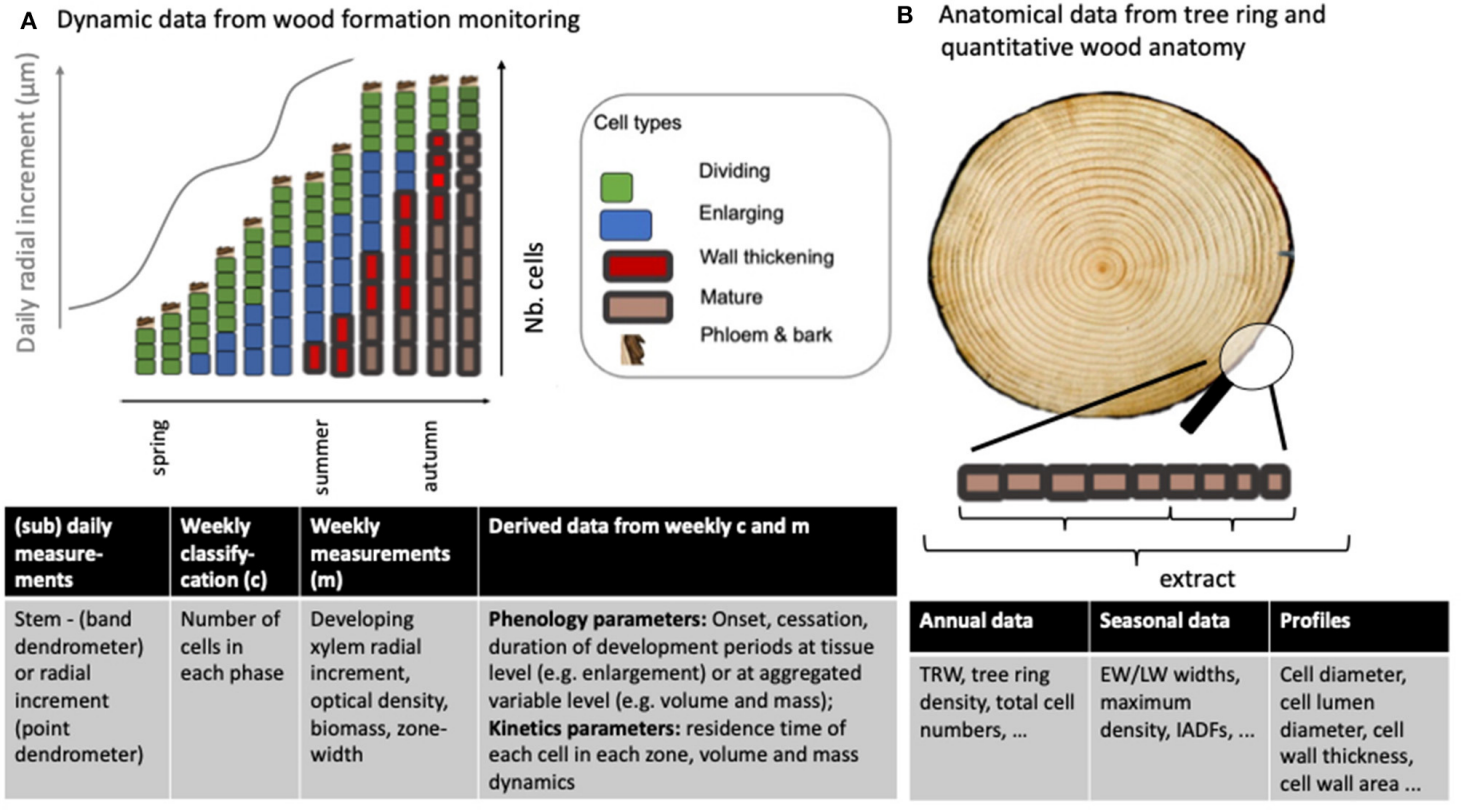
**(A)** Dynamic **(B)** static observations useful for wood formation model interrogation. **(A)**: (sub)-daily radial increment measurements are taken using dendromenters. Weekly classification requires staining methods, light microscopy and a human to identify and count cells of a given type. Weekly measurements can be semi-automated and do not necessarily involve the identification of specific cell phases. Weekly cell counts and measurements can be used to derive observations such as a period of presence/absence of a cell type (at the xylem tissue level) or the residence time of each cell in each phase (at the cell level, but also possible to derive at the tissue level). Tree disk image from Cuny et al. (2014).

Together with established types of observations, new sources of data have emerged against which models can be directly compared. These are not fully exploited today. The following section reviews two categories of data that have been used for model validation. We make a case that these and novel observations, as well as the combined use of observations, could be more commonly applied for model parameterisation and verification in order to increase our understanding of the mechanisms that drive xylogenesis. Observations related to wood formation (Figure 4) can be divided into 1) static data, which are end-of season observations of anatomical properties of mature cells or the ring itself (e.g., TRW, cell wall thickness, density profile) and 2) dynamic data, such as xylogenesis monitoring data from which we obtain snapshots on the number of cells in a given phase at the time of sampling. Both types of observations have deficits, but when used in tandem can supplement each other: Anatomical data originate through the process of xylogenesis, but the timing of individual processes cannot be reliably retraced from the data. In contrast, dynamic data can tell us about the kinetics of cell differentiation, but cell anatomy such as cell sizes or wall thicknesses cannot be inferred, as often the sampling distorts the true cell dimensions (e.g., the pressure applied to the still delicate cambial and enlarging cells during microcoring using a Trephor (Rossi et al., 2006a; but see Uggla et al., 1996). Observations can also be divided into data-sparse (e.g., the date of the start or the end of the enlargement process, the tree-ring width) and data-dense (e.g., weekly xylogenesis data, dendrometer data or intra-ring profiles of cell dimensions) observations. Wood formation models have been able to generate one or multiple types of output against which they can be compared with observations, depending on their aim and structure (see Table 2). Importantly, one must distinguish between using data for model development, parameterisation, and validation: the same data should not be used in all three instances.

**Table 2:** ⊕ model output compared against observations, ∅ (possible) output but not compared against observations. † Possible output but not reported. () model output, but created using an empirical relationship with previously modeled outputs. *Microdensity profile derived from wall thickness. Wilkinson et al. (2015) used the model by Deleuze and Houllier (1998), to simulate wall thickness rather than mass increase and could therefore resolve and compare against microdensity (see second ⊕*). CAM, cambial cells; ENL, enlarging cells. Note that being able to resolve xylogenesis, enables phenological events (e.g., start of CAM, Start /end of ENL, etc). Note that some models display output, which are not listed here, eg. maximum density, mean density, microfibril angle. Anatomical output related to wall thickness can be expressed in cell position (Hölttä et al., 2010) or as proportion of annual ring (%) (Drew and Downes, 2015), which is not distinguished in this table. Radial diameter can refer to either cell or lumen radial diameter. Tree ring width is equivalent to the end-of season value of cumulative radial growth, measured as cumulative cell anatomy properties or directly as ring width. Cell numbers is equivalent to end of season cumulative tracheid production. Xylogenesis refers to cell numbers or cell production rates derived from xylogenesis observations. TRWi, Tree ring width index.

**Model**	**TRW**	**Cell numbers**	**Density profile**	**Wall thickness**	**Radial diameter**	**Xylogenesis**
Wilson and Howard (1968)	∅	⊕	∅	⊕	⊕	
Howard and Wilson (1972)	∅	⊕	∅*	⊕	⊕	⊕
Wilson (1973)	∅	∅			⊕	⊕
Fritts et al. (1991)				()	⊕	
Deleuze and Houllier (1998); Wilkinson et al. (2015)	∅	∅	⊕⊕*	∅	⊕	
Fritts et al. (1999)						
Vaganov et al. (2006)	TRWi	⊕				∅ (CAM)
Drew et al. (2010)		∅	∅	∅	∅	∅
Hölttä et al. (2010)	∅	∅		∅	∅	∅
Drew and Downes (2015)	∅	∅	⊕	⊕ (mean) ⊕	⊕ (mean) ⊕	†
Schiestl-Aalto et al. (2015)	⊕	⊕				⊕
Hartmann et al. (2017)	†	†			∅	⊕ (CAM, ENL)
Cartenì et al. (2018)	†	†		⊕	⊕	
Hartmann et al. (2021)	⊕	†	⊕		⊕	⊕
Friend (2020)	∅	∅	⊕		∅	
Cabon et al. (2020)		∅				⊕ (CAM)

#### 2.6.1. Static Observations

Some of the most common static variables which wood formation models try to replicate are end of the year observations of ring width (e.g., Friend, 2020) or ring width index, (e.g., Vaganov et al., 2006) cell numbers (e.g., Vaganov et al., 2006; Friend, 2020) or wood intra-ring density profiles (e.g., Deleuze and Houllier, 1998; Drew and Downes, 2015; Friend, 2020). A hitherto unused type of static observations for wood formation model parameterisation or validation are isotope ratios (but see Tolwinski-Ward et al., 2015, which we however do not count as a wood formation model in this review).

Static observations differ in the extent to which they can validate a wood formation model or its individual processes. Firstly, models can be validated against data-sparse, single-point tree-ring parameters e.g., width, wood density, isotope ratio. While the former two observations are very abundant, the downside of only relying on this type of observation is that this involves the fitting of complex models to a single annual data point (e.g., TRW). This means for wood formation models that many different hypotheses will be able to replicate this type of observation through overfitting. Secondly, more data-rich static observations offer a higher spatial resolution for model validation. For example the final structure within the tree ring, such as its density profile can resolve intra-annual dynamics to some degree. Some wood formation models (Deleuze and Houllier, 1998; Fritts et al., 1999; Drew and Downes, 2015; Friend, 2020) simulate wood density profiles and compare their output against density profile observations. Treering3 (Fritts et al., 1999) can even automatically interface (code written by Geoffrey M. Downes) with the SilviScan digital output for model verification. SilviScan is a semi-automated device to rapidly obtain wood density observations (amongst others) using X-ray technology and image analysis. It was originally developed in 1992 for commercial forestry (Evans et al., 1994) and, after several upgrades, is still widely used in the scientific community. Nevertheless, while at a higher resolution, these static observations do not allow for the exact inference on the timing of the inception of these high and low-density features. Therefore, care is needed when applying a distance-to-time conversion approach across tree ring anatomical features (i.e., equally-sized sections of the ring have not emerged during an equally-long period (Pérez-de-Lis et al., 2021). This makes the attribution to environmental events from anatomical features alone difficult (but see Drew and Downes (2009) for how the additional use of dendrometers can address this issue to some degree). An exception is a specific type of data-rich static observations of cell anatomy, so-called intra-annual density fluctuations (IADFs). IADFs are unusual variations in cell size and wall thickness along a tree ring (Battipaglia et al., 2016). These density fluctuations can either be caused by earlywood-like cells in the latewood section of a tree ring or latewood-like cells in the earlywood section of a tree ring. Age or width of the rings can also play a role in the absence/presence of IADFS under IADF-conducive environmental conditions (Rigling et al., 2011). Both phenomena have been associated with precipitation after a summer drought (earlywood-like IADFS) (Campelo et al., 2007; Rigling et al., 2011), or the absence of precipitation during early spring (latewood-like IADFS) (Wimmer et al., 2000). Thus, the resulting signal in the cell anatomy can be related back to a specific period during the growing season. However, there are many open questions as to the mechanisms that cause IADFs (see Battipaglia et al., 2016 for a good overview), and it is still unclear what cell developmental phase is affected to cause these deviations from the common anatomy. Therefore, these observations are especially valuable for the validation of wood formation model hypotheses. Similarly, wood formation models can help explore which cell processes are the most likely to be affected. IADFs comparison with model-simulated IADFs has far only been done by Wilkinson et al. (2015) who applied the model from Deleuze and Houllier (1998) and could indeed replicate IADFs at a water-limited site. Overall, static observations, with some exceptions through IADFs, cannot be fully be used to reconstruct the timing and thus environmental conditions of wood formation processes occurring intra-annually.

#### 2.6.2. Dynamic Observations

This issue of static observations can be overcome when using dynamic xylogenesis observations. Generating dynamic observations typically involves the weekly sampling of the growing ring, to derive weekly cell counts of each cell type within a differentiation phase, or (more common for angiosperms) the width of each developing zone. Models which have used xylogenesis observations to some degree are Cabon et al. (2020) for cell production, Hartmann et al. (2017) and Hartmann et al. (2021) for cambial and enlarging cells and Schiestl-Aalto et al. (2015) for all cell types. While some models are able to produce xylogenesis output (e.g., Fritts et al., 1999, see Table 2), they do not compare it against data (but see Schiestl-Aalto et al., 2015). Instead, they discuss qualitatively the shape of the observed cell numbers. Xylogenesis observations can be data-rich, if sampled frequently, across many trees, throughout the growing season. One issue with xylogenesis data is the between-tree variability in the dynamics, which so far have been addressed through normalization approaches, e.g., by standardizing against the total number of cells of the previous year Rossi et al. (2003) to fit gomperts or general additive models Cuny et al. (2013) to cell production observations. Besides for phenological purposes, such as determining onset or cessation of wood formation (critical dates), these observations can be directly related to co-occuring environmental conditions, which is useful for increased process-understanding related to environmental factors acting upon different cell types. Other data-rich observations of stem radius variations are dendrometer-data, which, while temporally very fine-grained (i.e., tens of minutes), are however impossible to interpret when it comes to disentangling which cell phase (cambial or enlarging) contributes to the observed growth increment and are therefore more useful to verify wood formation models' overall increment dynamics. Nevertheless, dendrometers are essential tools to determine the critical sub-daily time periods during which growth variations actually occur and which environmental factors matter. For example, Zweifel et al. (2021) determine that wood radial growth is most likely to occur during the night or at dawn, when vpd is low. Their findings make clear that daily aggregation of environmental variables to drive wood formation models must be done with care. To our knowledge, dendrometer data has so far not been used for wood formation model validation, rather for radial growth or stem increment models. Therefore, we see scope for involvement of this type of observation to help fill the gap between cell counts at weekly time-scales with daily-resolved “anatomical” information on radial increment. Nevertheless, challenges remain to attribute observed increments to irreversible growth due to diurnal shrinking and swelling of the stem (but see Mencuccini et al. (2017) and Zweifel et al. (2016)) and to account also for phloem growth dynamics.

New types of observations continue to be developed which are able to enhance inter-species comparison and monitoring of variables emerging from xylogenesis dynamics such as volume and mass variables, also relevant for wood formation model validation. For example, zone width information from weekly microcores, rather than cell count, is less time-consuming and may enhance comparison across species (e.g., angiosperms vs. gymnosperms): instead of counting cells week^−1^, one only measures the weekly zone width of a certain type of cells (e.g., see Prislan et al., 2019) (e.g., enlarging and thickening, mature cells). While the latter zone-width approach is more coarse, it is common practice in angiosperms, which have to overcome increased complexity by more cell types developing, such as large vessels, that can make it hard to objectively count a single radial file (as depicted in Figure 1). An angiosperm-gymnosperm comparison of xylogenesis dynamics using zone-width observations has so far only been done by Martinez del Castillo et al. (2016). Another study has used Norway spruce *(Picea abies (L.) Karst)* to investigate a novel histological approach that only monitors the dynamics of volume or mass increase (Andrianantenaina et al., 2019) as opposed to the conventional cellular-based approach which monitors cell developmental stages separately. The monitoring of volume and mass variables only are potentially useful for parameterizing and verifying parsimonious wood formation models useful for regional-scale to global modeling, for example in new generations of DGVMs. Recently a new method called high-resolution X-ray computed tomography (HRXCT) is also capable of monitoring intraannual stem radial width (called “size growth” by the authors), and biomass dynamics and promises to create further such observations relevant to modeling wood formation (Lehnebach et al., 2021). However, whether or not all information content necessary for model validation can be retained by all these new types of observations remains unclear, as neither zone-width nor volume and mass- only data have so far been used to validate any wood formation models. While cell numbers have been used for model validation, neither zone-widths, nor volume and mass-based approaches have hitherto been exploited in wood formation modeling.

Overall, the use of intra-ring (especially cell anatomical) and xylogenesis data in tandem will likely provide the best way to challenge individual model process hypotheses around each cell developmental phase, its drivers, and the resulting anatomical features. The only model which to our knowledge has formally compared output against both dynamic (xylogenesis) and static (cell anatomical) data is XyDys1 and 2 (Hartmann et al., 2017, 2021). The challenge remains to integrate dynamic and static data in the light of a lot of sample variability. While significant progress has been made in this from the data analysis side (e.g., Cuny et al., 2013), wood formation models will be a useful bridge between the datasets.

Not discussed in any detail in this review are molecular-level and gene expression observations, which remain unused in wood formation model verification or hypothesis construction, with the exception of auxin and sugar. Whether statistical association of small genetic mutation with observed traits, currently mostly used for molecular breeding (e.g., reviewed by Du et al., 2018), gene expression level analysis across the developing wood and in response to hormonal changes (e.g., Schrader et al., 2004; Immanen et al., 2016), or the construction of knockout tree variants to obtain functional understanding through the artificial absence of a protein in a crucial process within wood formation (e.g., Xu et al., 2021), all knowledge and data generated from these methods are additional powerful resources that can be harnessed for wood formation model development and verification in the future.

#### 2.6.3. Model-Data Interoperability Through Data Standards and Analysis Tools

Data standards help both modelers and experimentalists make their research output interoperable among each other. Recently developed data-analysis tools such as CaviaR (Rathgeber et al., 2018) can help clarify concepts such as critical dates (of when enlargement or wall thickening begin and end) and provide a standard format in which to handle wood formation observations. These data analysis tools offer opportunities for modelers to develop similar-looking “virtual tree” output, thus facilitating model-data comparison. Whereas, Fritts and Shashkin (1995) were impeded by the lack of image analysis tools and therefore slow sample processing, today, image analysis tools such as WinCELL (Regent Instruments, 2012), ROXAS (von Arx and Carrer, 2014), or ImageJ (cf. Schuldt et al., 2013 for user-example) can be used to study tree ring anatomical structure including cell diameter, lumen area, or wall thickness amongst others. These tools provide valuable smaller-scale data which can be used to confront models. Their semi-automated nature can provide large datasets to verify models against. When used together with dynamic xylogenesis observations they have great potential to constrain wood formation models and shed more light onto their plausible structures.

Overall, the use of data should help verify wood formation models further. The utility of the data depends on the model, the processes it resolves and the purposes it serves. However, in general the most useful combination of datasets for model validation are a combination of both dynamic (xylogenesis) and static (anatomical) data. Observations can both be used for model hypothesis validation, but there is unused potential to also apply it to model calibration. An enhanced integration between data and model output through shared formats will facilitate direct model–data comparison. Ultimately, model development and gathering of observations and their standardized analysis should go hand-in-hand to generate new knowledge.

### 2.7. Wood Formation Under Climate Change

That wood growth will be impacted by climate change is already evident (e.g., Briffa et al., 1998; Pretzsch et al., 2018; Babst et al., 2019). However, only few wood formation models have been originally built to investigate climate change impacts on tree growth as principle motivation. Indeed, few wood formation modeling studies even mention the importance of wood formation modeling to predict growth responses in a climate change context. The exception is Drew and Downes (2015), who point out the suitability of their model to better predict future forest productivity. In the preface to their book, Vaganov et al. (2006) mention a potential for global carbon cycle modeling, but the ultimate model focus of the VS-model is extracting historical climate patterns. Nevertheless, the VS-model has been used to reconstruct and forecast growth phenology from climate forcings and TRW data at the Tibetan Plateau and it was found that growth start has shifted forward by 6 days (Yang et al., 2017), and that growing season length will continue to increase with climate change (He et al., 2018a,b). However, it is important to note that while these authors found a prolonged period of potential growth which could be associated with increased TRW and thus carbon sequestration, climate change-induced changes in phenology cannot be universally seen as cause for increased tree growth (Körner and Basler, 2010). Therefore, not only the growing season length but within-season processes must be addressed using wood formation models. Nevertheless, there is potential in improving the mechanisms related to wood formation phenology in the models: A chilling-influenced heat sum model performed best in an intercomparison of approaches for simulating the onset of enlargement in developing wood (Delpierre et al., 2018), an interesting mechanism to test in wood formation models. Predicting the end of wood formation processes, e.g., cessation of cambial activity (Buttò et al., 2020b) or until full maturation (Cuny et al., 2019) has hitherto been difficult and more research is needed in this area. Since wood is a large and long-term carbon store, and wood formation dynamics can help predict carbon allocation in trees (Buttò et al., 2021), wood formation models should be integrated to study global carbon cycle dynamics. However, due to the lack of wood formation models suitable for global use, their application in global carbon cycle and vegetation modeling are currently lacking (Friend et al., 2019).

The tree ring and wood formation community has started to encourage the use of wood formation and tree ring observations for the global modeling of wood formation (Babst et al., 2014, 2018; Zuidema et al., 2018). Some vegetation modelers have also started work to this end (Friend et al., 2019; Friend, 2020; Eckes-Shephard et al., 2021). This new area of modeling offers a new application of wood formation models, namely to help improve predictions on global vegetation carbon responses to climate change.

Hitherto unexplored areas in wood formation modeling is the growth response to wind sway and nutrient availability. Nutrients were not important in previous study contexts and there is large uncertainty in how to represent these additional processes. For a global wood formation model, this is an important area for further research, as global productivity, especially in forests, has commonly been found to be nutrient limited (LeBauer and Treseder, 2008; Fernández-Martínez et al., 2014). It has also been shown that macro nutrients may impact wood density and timber stiffness in fast grown pines (see e.g., Wessels et al., 2015). Especially for new areas of applications of wood formation models, e.g., global modeling, nutrients may need to be considered to some extent. It is important that the coverage of observations of wood formation dynamics, anatomy, and tree rings increases in low-latitude areas in order to inform the development of globally-applicable wood formation models.

## 3. Conclusion and Outlook

This review has shown how wood formation modeling, from the pioneering efforts in the 1960s to today, has greatly improved our mechanistic understanding of wood formation. We have highlighted areas where existing wood formation hypotheses may need to be challenged. There is significant scope for exploring new hypotheses and to better integrate them within the models. There is great potential for collaboration between researchers performing long-term field monitoring (e.g., Integrated Carbon Observation System (ICOS)), experimentalists (e.g., Free Air Carbon Enrichment (FACE) and greenhouse experiments) and modelers to address outstanding questions. We envision that wood formation modeling can help to address key challenges related to global change and carbon cycle modeling.

We have summarized the current knowledge of growth process representation in wood formation models. Researchers from three disciplines have developed 17 wood formation models at various levels of detail and with different assumptions on environmental drivers and applications in mind. While dendroclimatologists are interested in the growth–climate relationships in order to reconstruct past climate from tree rings, foresters aim to predict wood quantity and quality. Finally, more fundamental researchers have built many models with the aim to better understand variability, hormonal influences, or growth-carbon interactions. Underlying all these models are a wide range of different hypotheses, supported by multiple lines of empirical evidence on what processes are necessary to resolve when modeling tree growth. The questions posed with the models very much determine their focus and level of complexity. It is therefore not surprising that the models differ substantially from each other. However, the fact that there is rather little agreement on some basic processes (e.g., the influence of hormones on wood formation; what causes the transition between earlywood and latewood; the influence of carbon supply), and their drivers (see Figure 3) shows that there is still a lot to be studied about wood formation, which manifests itself in uncertain wood formation models.

Wood formation models have already been successfully applied to answer many different scientific questions. Besides their current remit, they have the potential to be useful in many other areas. For example, simple wood formation models may be useful for global application to better project vegetation carbon responses to the environment and hence climate change (Friend et al., 2019). For this, understanding the role of carbohydrates on wood formation (regulatory or as substrate and at what developmental process is it restricting) will need to increase for example through manipulation experiments (e.g., Rademacher et al., 2019). Additionally, simulating future tree rings could help forecast tree mortality in conjunction with tree ring-based mortality algorithms (Cailleret et al., 2017). Finally, resolving growth processes as a carbon sink within the tree may help to answer questions on active vs. passive storage and can in the same context also help address the source-sink controversy (Schiestl-Aalto et al., 2015).

Data sources to verify growth hypotheses within the models are far from fully exploited. Most models compare their output against end-of-the-year observations such as density, ring width, number of cells, or mean tracheid diameter. Dynamic data such as xylogenesis data can help verify whether the intra-annual dynamics are indeed captured well in those models. While many different model hypotheses may be able to replicate a final-year result well, this finer-grained data is important for challenging model hypotheses on a shorter time-scale, and hence addressing mechanisms more precisely. Additional end-of-season output that may also be more challenging for wood formation models to replicate are IADFs, which in tandem with xylogenesis data deserve more attention from wood formation modelers. Future efforts should also make use of molecular studies for hypothesis building or model verification.

This review identified three main areas (carbon, hormones, and more broadly, or as a result earlywood-latewood transition) where model hypotheses diverge and therefore on which additional research should be done. However, while wood formation seems to be subject to multiple internal and external controls simultaneously, observations *in natura* may not always provide conclusive evidence toward one mechanistic hypothesis for a model. Therefore, we call for a move toward manipulation experiments (e.g., Baba et al., 2018; Rademacher et al., 2019) and combinations of anatomical data, IADFs, and weekly xylogenesis monitoring. From our summary of the current state of wood formation modeling research, we identified the following (inter-related) areas in which open questions remain:

(1) hormonal influences on growth
(2) carbon influences on growth. Addressing these two areas of research will already contribute to the outstanding mechanisms on
(3) earlywood–latewood transitions.

Global change will affect wood formation in all forested regions of the world and challenge the plausibility of existing hypotheses encapsulated in wood formation models. The modeling and wood formation observations are currently biased toward the northern hemisphere. Therefore, there is great potential in the wood formation modeling and observation community to increase their area of research into other low-latitude ecosystems. This, together with an increased use of diverse observations from multiple disciplines, will be crucial in verifying the hypotheses behind wood formation's mechanisms and drivers. Getting these right will be critical for all applications of wood formation hypotheses, for the single tree or global vegetation model.

## Data Availability Statement

The original contributions presented in the study are included in the article/supplementary material, further inquiries can be directed to the corresponding authors.

## Author Contributions

AE-S conceived the content of the review and drafted the manuscript with input from all authors. AF supervised the project. All authors commented on the final manuscript.

## Funding

AE-S acknowledges support from the European Research Council under the European Union Horizon 2020 Programme (grant no. 758873, TreeMort). FCL was supported by the Swedish Research Council (Vetenskapsrådet, grant no. 2018-01272) and conducted the work with this article as a Pro Futura Scientia XIII Fellow funded by the Swedish Collegium for Advanced Study through Riksbankens Jubileumsfond. This study contributes to the Strategic Research Areas BECC and MERGE. CR was supported by a grant overseen by the French National Research Agency (ANR) as part of the Investissements d'Avenir program (ANR-11-LABX-0002-01, Lab of Excellence ARBRE).

## Conflict of Interest

The authors declare that the research was conducted in the absence of any commercial or financial relationships that could be construed as a potential conflict of interest.

## Publisher's Note

All claims expressed in this article are solely those of the authors and do not necessarily represent those of their affiliated organizations, or those of the publisher, the editors and the reviewers. Any product that may be evaluated in this article, or claim that may be made by its manufacturer, is not guaranteed or endorsed by the publisher.
